# Ionic Organic Network-based C3-symmetric@Triazine core as a selective Hg^+2^ sensor

**DOI:** 10.1080/15685551.2024.2360746

**Published:** 2024-06-18

**Authors:** Maha A. Alshubramy, M. M. Alam, Khalid A. Alamry, Abdullah M. Asiri, Mahmoud A. Hussein, Mohammed M. Rahman

**Affiliations:** a Chemistry Department, Faculty of Science, King Abdulaziz University, Jeddah, Saudi Arabia; b Department of Chemical Engineering, Z. H. Sikder University of Science and Technology (ZHSUST), Shariatpur, Bangladesh; c Chemistry Department, Faculty of Science, Assiut University, Assiut, Egypt; d Center of Excellence for Advanced Materials Research (CEAMR), King Abdulaziz University, Jeddah, Saudi Arabia

**Keywords:** C3-symmetry, poly pyridinium, triazine, nanocomposites, GNPs, MWCNTs, mercury ion detection, electrochemical method, environmental safety

## Abstract

The C3-symmetry ionic polymer PPyTri has been designed with multi-walled carbon nanotubes (MWCNTs) or graphene nanoplatelets (GNPs) and studied as an ultrasensitive electrochemical sensor for trace Hg(II) detection. The synthesis approach incorporated attaching three pyridinium cationic components with chloride anions to the triazine core. The precursors, BPy, were synthesized using a condensation process involving 4-pyridine carboxaldehyde and focused nicotinic hydrazide. The polymer PPyTri was further modified with either MWCNTs or GNPs. The resulting ionic polymer PPyTri and its fabricated nanocomposites were characterized using infrared (IR), nuclear magnetic resonance (NMR), scanning electron microscopy (SEM), transmission electron microscopy (TEM), and powder X-ray diffraction (XRD). The analysis revealed that both the polymer and its nanocomposites have semi-crystalline structures. The electroactivity of the designed nanocomposites toward Hg + 2 ions revealed that among the nanocomposites and bare copolymer, the glassy carbon electrode (GCE) adapted with the PPyTri GNPs-5% exhibited the greatest current response over a wide range of Hg + 2 concentrations. The nanocomposite-modified electrode presented an excellent sensitivity of 83.33 µAµM − 1 cm − 2, a low detection limit of 0.033 nM, and a linear dynamic range of 0.1 nM to 0.01 mM (R2 = 0.9945).

## Graphical abstract

**Figure:**
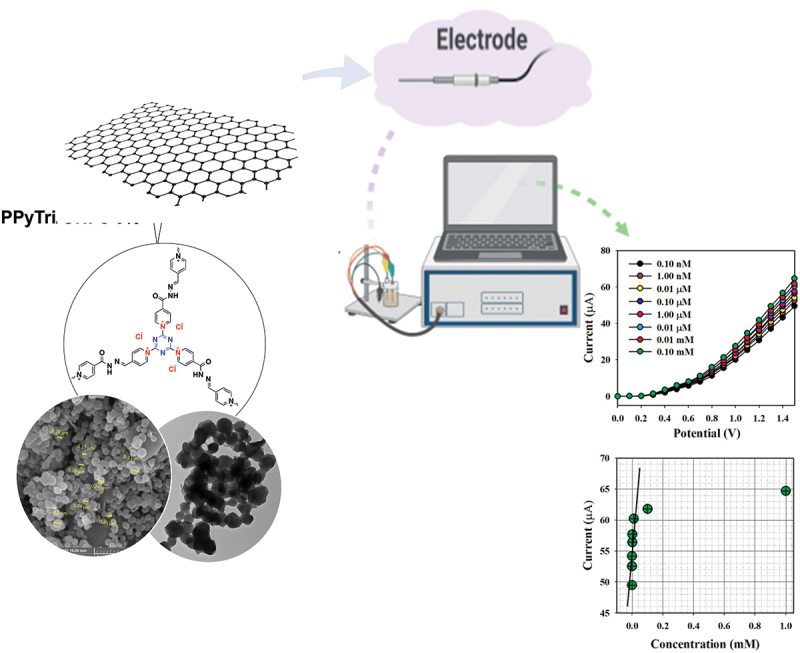

## 1. Introduction

Heavy metal toxicity is critically important due to its poisonous effects on human health and ecological systems. Among all heavy metals, mercury (Hg) is one of the most universal and harmful pollutants found in natural environments and results from both natural and anthropogenic sources [1]. Given its persistence in the environment, Hg is stored in aquatic ecosystems, water sources such as rivers, lakes, and oceans, and soil with high absorption in biological tissues and a slow elimination rate. The accumulation of Hg^+2^ ions is hazardous to humans and the environment, even at low concentrations. Scientific studies have revealed that the toxic impacts of Hg^+2^ ions can lead to various health issues in humans, such as damage to nervous and endocrine systems, adverse effects to the brain and kidneys, and conditions like Hunter – Russell, Alzheimer’s, and Minamata [2–6].

A reliable and efficient method is needed to better detect Hg^+2^ ions. Several methods, such as inductively coupled plasma mass spectrometry, chemiluminescence, fluorescence, and atomic absorption spectrometry, have been extensively used to confirm the presence of Hg^+2^ ions [7–10]. However, these methods have several drawbacks, such as high costs, complexity, bulky instrumentation, time-intensive processes, and impracticality for on-site measurements [11–14].

Nanoporous ionic organic networks (NIONs) are a versatile class of materials that have recently emerged as promising candidates for electrochemical applications. These have covalently tethered cations and/or anions within a nanoporous structure, enhancing adsorbate – absorbent interactions through polarization effects and Coulombic forces. NIONs are readily modified through ion exchange reactions and can influence charge transport mechanisms, enhance ion conductivity, and introduce exotic photoelectrochemical properties. High ion densities within a conjugated skeleton indicate NIONs are a valuable structure for engineering ‘smart’ materials with tailored electrochemical responses [15–34].

In parallel, materials with C3-symmetry have a unique structural platform that makes them suitable for various applications. These materials possess a three-fold rotational symmetry that impacts their electronic and optical properties. In electrochemical applications, materials with C3-symmetry provide a specific environment for incorporating functional moieties, such as triazine and pyridinium groups [35–37]. Triazine and pyridinium moieties are known for their electrochemical activity and stability, making them ideal candidates for use in nanoporous materials. The triazine ring structure is stable and promising in facilitating electron transfer processes. Similarly, pyridinium moieties exhibit redox activity, enabling reversible electrochemical reactions. Combining these moieties in nanoporous materials with C3-symmetry can create tailored electrochemical interfaces with various applications from energy storage to sensing [38–42].

The development of nanoporous materials is crucial for enhancing electrochemical performance. Multi-walled carbon nanotubes (MWCNTs) and graphene nanoplatelets (GNPs) are popular additives due to their unique properties. Integrating these nanomaterials improves composite conductivity, mechanical strength, and electrochemical stability. Combining organic networks with carbon materials enhances electron transfer, meeting electrochemical sensing requirements. Carbon nanoplatelets are widely used for their conductivity. Studies have shown that combining organic networks with carbon materials significantly enhances electrochemical performance [43–45]. For example, Zhixiang Xu et al. [46] modified a glassy carbon electrode with a graphene organic polymer composite, resulting in increased peak current and electroactive surface area. Similarly, Dawei Pan et al. [47] synthesized organic networks with carbon materials, leading to excellent electrochemical performance. These findings validate the effectiveness of using organic networks/carbon materials for superior electrochemical sensors.

Thus, this study explores the synergies between nanoporous ionic organic networks, C3-symmetry materials, and electrochemical functionalities conferred by triazine and pyridinium moieties. Additionally, we investigate the impact of nanomaterial fabrication techniques, particularly the use of MWCNTs and GNPs, on enhancing the electrochemical performance of these materials. The **PPyTri/GNPs** or/**CNTs** were prepared and modified over a glassy carbon electrode (GCE) and studied for their electroactivity toward Hg^+2^ ions. The electrochemical performance of the sensor was thoroughly assessed in a phosphate buffer medium and found to be extremely sensitive and selective for Hg^2+^ ions.

## 2. Experiments

### 2.1. Materials and equipment

All solvents and chemicals were analytic grade and purchased from Sigma Aldrich. The MWCNTs and GNP were purchased from Aldrich Nano Tech Co. LTD. Egypt. Analytic grade inorganic salts Cu^+2^, Ni^+2^, Cd^+2^, Sn^+2^, Pb^+2^, Co^+2^, Hg^+2^, and Zn^+2^ were obtained from the supplier as required. All chemicals were used as received without further purification. Fourier transform infrared (FT-IR) spectra were recorded using a PerkinElmer Spectrum 100 FT-IR device in the range of 4000–500 cm^−1^. The morphologies and elemental distributions of the polymers were examined using scanning electron microscopy (SEM, TESCAN VEGA 3, Czech Republic). The samples were mounted on aluminum microscopy stubs using carbon tape and coated with gold (Au) for 120 s using a Quorum Techniques Ltd sputter coater (Q150t, UK). A JEOL JEM 1400 plus transmission electron microscope (TEM) investigated interactions between the polymers and nanomaterials. X-ray diffraction (XRD) data were collected using a Bruker D8 Advance X-ray diffractometer operating at 40 kV and 25 mA with a Cu Kα irradiation source. An electrochemical cell was constructed using a Keithley Electrometer to perform electrochemical (I-V) analysis.

### 2.2. Synthesis of Schiff base

The Schiff base **BPy** was synthesized from the condensation reaction of 4-Pyridine carboxaldehyde (1 mmol) with nicotinic hydrazide (1 mmol). The mixture was dissolved in ethanol as a solvent with drops of concentrated HCl (37%) as a catalyst. The reaction mixture was equipped under a reflux system and stirred for 3 h at 70 °C. The obtained Schiff base was filtered, washed thoroughly with ethanol, and dried under vacuum. The recrystallization process used acetone to provide the pure Schiff base as white crystals (90%). **FTIR**: ν [cm^‒1^]: 3199 (Ar C-H stretch), 1684 (C=O, amide), 1599 (C=N, imine), 1567 (C=N, pyridine), 1412 (Ar C=C bending), 1287 (Ar C=N bending), 1149 (Ar C-N bending), and 827 (Ar C-C bending). ^**1**^**H NMR** (850 MHz, DMSO-*d*_*6*_): δ_H_ = 7.00 (d, 4 H, Ar-**H**), 7.09 (d, 4 H, Ar-**H**), 7.75 (s, 1 H, **H**C=N), 7.93 (d, 4 H, Ar-**H**), 8.03 (d, 4 H, Ar-**H**), and 11.78 (s, N**H**). ^**13**^**C NMR** (200 MHz, DMSO-*d*_*6*_): δ_C_ = 124, 126, 142, 145, 149, 152, 153, and 165.

### 2.3. Synthesis of PPyTri polymer

Cyanuric chloride (6.0 mmol, 1 eq) was dissolved in 30 mL ethyl acetate and added dropwise into a stirred solution of **BPy** (9.0 mmol, 1.5 eq) in 50 mL of ethyl acetate. Then, the reaction mixture was refluxed and stirred for 48 h. The obtained precipitate was then filtered and washed with ethyl acetate three times before drying in a vacuum, giving the product **PPyTri** as a light green powder (yield: > 90%). **FTIR**: ν [cm^‒1^]: 3199 (Ar C-H stretch), 1684 (C=O, amide), 1599 (C=N, imine), 1567 (C=N, pyridine), 1412 (Ar C=C bending), 1287 (Ar C=N bending), 1149 (Ar C-N bending), and 827 (Ar C-C bending). ^**1**^**H NMR** (850 MHz, DMSO-*d*_*6*_): δ_H_ = 7.43 (m, 4 H, Ar-**H**), 7.86 (m, 4 H, Ar-**H**), 7.05 (s, 1 H, **H**C=N), 8.12 (d, 4 H, Ar-**H**), 8.52 (m, 4 H, Ar-**H**), and 12.66 (s, N**H**). ^**13**^**C NMR** (200 MHz, DMSO-*d*_*6*_): δ_C_ = 129, 131, 133, 136, 137, 145, 150, 153, 154, 156, 157, 166, and 170.

### 2.4. Preparation of nanocomposite PPyTri polymer with GNPs or MWCNTs

A series of polymers with GNPs or MWCNTs were prepared via *in-situ* polymerization using different loadings (2 and 5%) of MWCNTs or (2 and 5%) of GNPs with respect to the unmodified copolymer. For each formulation, a mixture of 2 and 5% of MWCNTs or GNPs in H_2_O was added to equimolar amounts of the two monomers **CC** and **BPy**, and sonication was continued for 30 min followed by stirring for 16 h in ethyl acetate at 100 ºC. The process of analyzing the newly formed nanocomposites was similar to that of the bare copolymer nanocomposites as **PPyTri/CNTs-5%, PPyTri/CNTs-2%, PPyTri/GNPs-5%**, and **PPyTri/GNPs-2%**.

### 2.5. Fabrication of GCE with PPyTri/CNTs and PPyTri/GNPs nanocomposites

The I-V electroanalytical method modified the desired electrochemical sensor on the surface of a GCE with **PPyTri/CNT** or **GNP** nanocomposites. A thin homogenous layer of **PPyTri/CNTs** or **GNPs** was prepared in ethanol and deposited onto a GCE with a surface area of 0.0316 cm^2^. A drop of Nafion (5% Nafion suspended in ethanol) was added to the modified electrode after the drying step to obtain the desired stability. The GCE was subsequently subjected to thorough drying in an oven at 35 °C for an appropriate duration. An electrochemical cell was constructed using a Keithley electrometer, wherein either **PPyTri/CNTs/binder/GCE** or **PPyTri/GNPs/binder/GCE** was utilized as the working electrode, and a plain Pt wire was used as the counter electrode.

Then, a mercury (II) ion solution was prepared and used as the objective analyte. Linearity was established from the relationship between the current and concentration of Hg^2+^ ions, resulting in a calibration curve. Its slope assessed the fabricated sensor’s sensitivity and detection limit (DL). Calculating the linear dynamic range (LDR) involved determining the calibration curve’s maximum linearity (R2). The electrochemical investigation comprised maintaining a constant volume at 10.0 mL of a phosphate buffer solution in the detection beaker throughout the experiment. The electrochemical sensor utilized in this study employed a Keithley electrometer and featured a straightforward two-electrode system (working and counter electrodes).

## 3. Results and Discussions

### 3.1. Chemistry

The synthesis of C3-symmetry polymers requires considering the core structure and linkers. New ionic polymers with a C3-symmetry structure were created and evaluated for their electrochemical performance toward Hg^+2^ detection. The polymers incorporated a triazine core, viologen linkers, and MWCNTs or GNPs, improving their electrostatic and aromatic properties. This process enhanced their overall behavior using the electrochemical method.

### 3.2. Synthesis and characterization of Schiff bases and pure ionic copolymer (BPy, PPyTri)

**Scheme 1.**
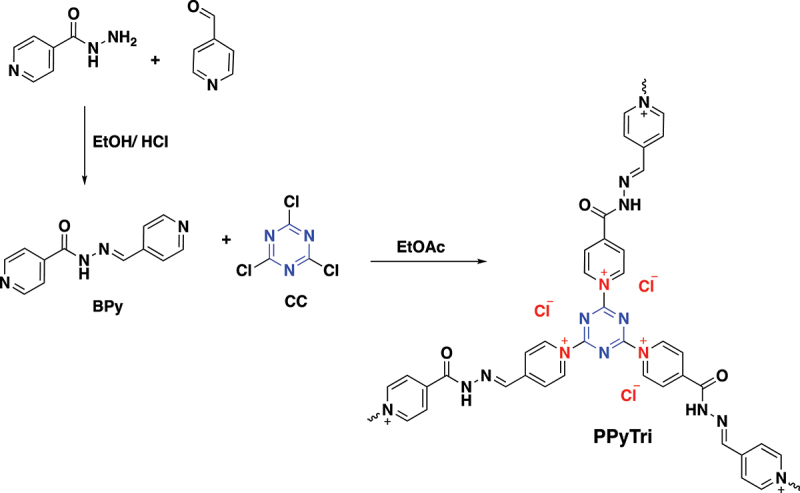
Systematic illustration to synthesize monomers **BPy** and polymer **PPyTri**.

**Scheme 2.**
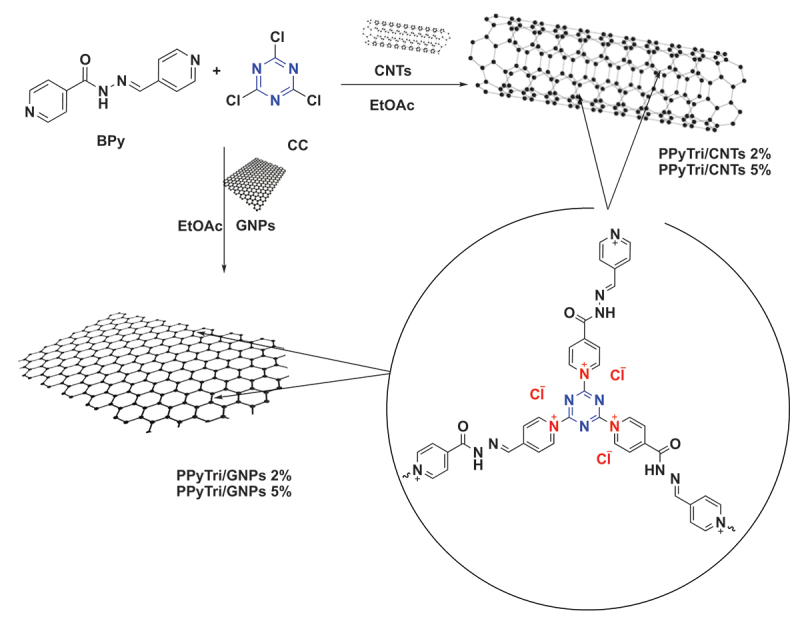
Systematic illustration of nanocomposite fabrication for **PPyTri/CNTs-5%**, **PPyTri/CNTs-2%**, **PPyTri/gnps-5%**, and **PPyTri/gnps-2%**.

The synthesis of a pyridinium ionic polymer **PPyTri** (possessed C3-symmetry) and its nanocomposites was achieved through the synthetic method shown in Schemes **1** and **2**. In the first step, the precursor Schiff bases **BPy** were synthesized by thermally condensing 4-pyridine carboxaldehyde with nicotinic hydrazide in refluxing ethanol with a catalytic amount of acid (Scheme **1**). The following step involved the Menshutkin reaction, widely used for producing polymers with quaternary pyridinium and ammonium salts. As illustrated in Scheme **2**, one pot polymerization process occurred between cyanuric chloride as the core of the C3-symmetry construction with the synthesized Schiff bases **BPy** as a linker to form the desired cationic polymer **PPyTri**, where chloride plays the counter anion role.

**Figure 1.**
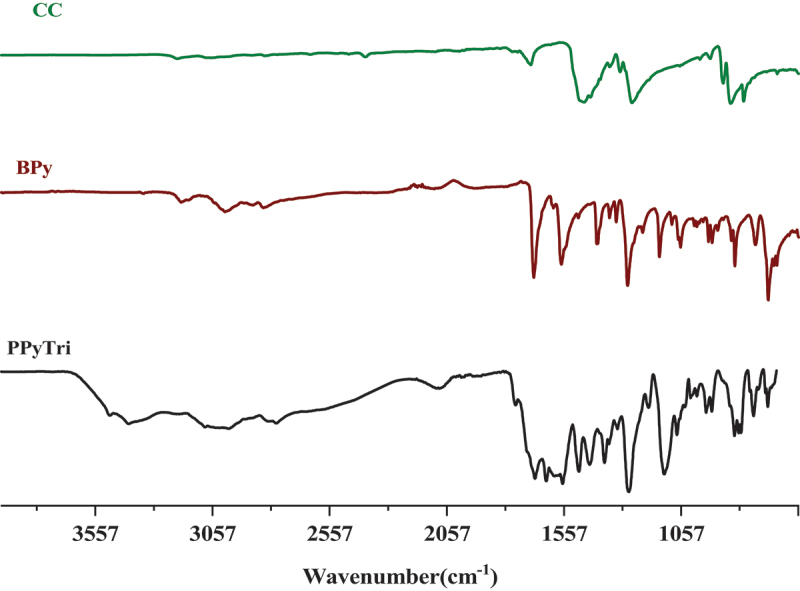
FT-IR spectra for monomers **BPy** and **CC**, and the polymer **PPyTri**.

**Figure 2.**
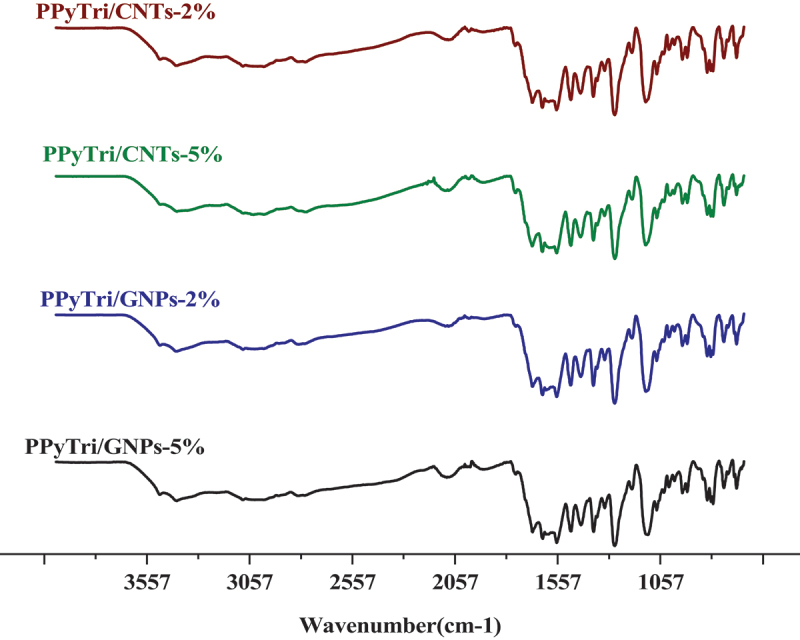
FT-IR spectra for nanocomposites.

The success of synthesizing the targeted C3-symmetry ionic polymers and their corresponding monomers was supported by investigating their spectral data from the FT-IR and NMR results. The FT-IR spectra of the polymer **PPyTri** (Figure 1(A)) showed the disappearance of the stretching vibration near 850 cm^−1^, attributed to the **C-Cl** band of their cyanuric chloride and supporting the quaternization of the pyridine ring in the monomer **BPy**. A new intense band belonging to pyridinium cations was observed near 1645 cm^−1^, further evidence of the successful quaternization reaction and the targeted ionic polymer PPyTri formation. The spectra also showed a shift in the C=N absorption bands of the triazine ring (1508 cm^−1^) compared to their precursor **CC**. The C=O amide linkage and C=N absorption of the imine and pyridine rings were observed at 1684, 1599, and 1567 cm^−1^, respectively.

### 3.3. Preparation and characterization of nanocomposites (PPyTri/CNTs and PPyTri/GNPs)

The fabrication procedure of bare copolymer incorporated loadings at various MWCNT or GNP ratios. The series of nanocomposites, **PPyTri/CNTs-5%, PPyTri/CNTs-2%**, **PPyTri/GNPs-5%**, and **PPyTri/GNPs-2%**, were produced through *in-situ* polymerization with a similar execution as the bare copolymer, as in Scheme **2**. The distribution of the loaded nanostructure was reinforced through an ultrasonic technique [46]. Ultrasonic irradiation is a versatile, eco-friendly, and straightforward approach for synthesizing nanostructured materials that are typically difficult to produce using conventional methods. Pressure oscillations are generated by subjecting liquids to ultrasonic waves, resulting in cavity formation. These cavities are effectively employed in homogenization to achieve uniform nanoparticle distributions throughout the final product. The nanocomposites were analyzed utilizing FT-IR spectroscopy, and their surfaces were examined using SEM, XRD, and TEM.

**Figure 3.**
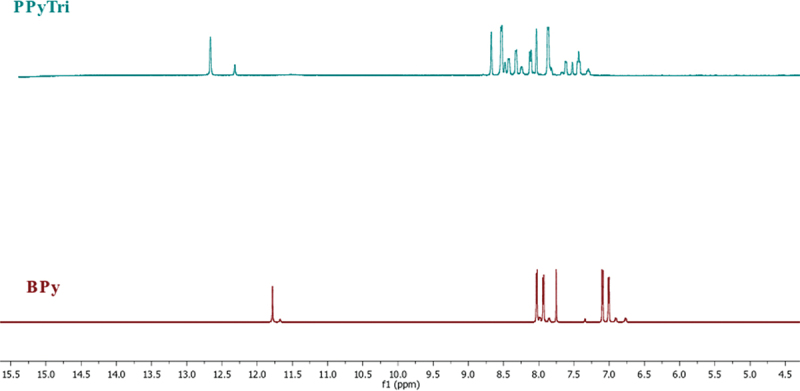
[1]H NMR spectra for the monomer **BPy** and polymer **PPyTri.**

**Figure 4.**
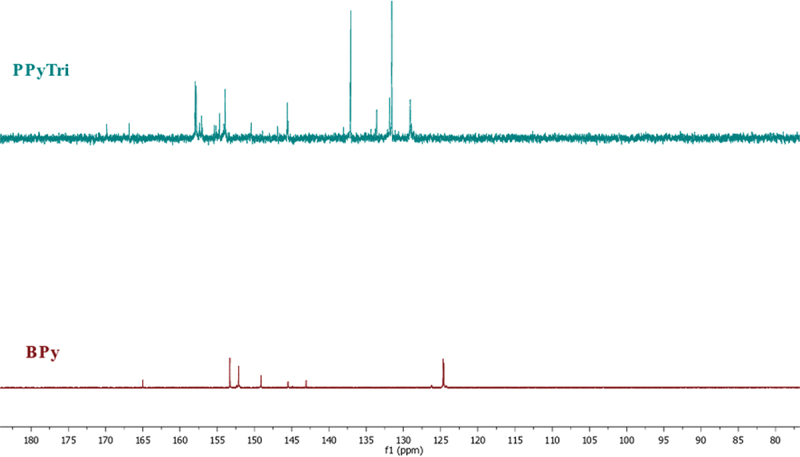
[13]C NMR^1^H NMR spectra for the monomer **BPy** and polymer **PPyTri**.

The FT-IR spectra of the created nanocomposites are presented in (Figure 2). In the spectra, the band at 1612 cm^−1^ is assigned to the C=C stretching vibration of either MWCNTs or GNPs, while the broad peak at 1530 cm^−1^ is attributed to C-C plane vibrations of the graphitic walls of either MWCNTs or GNPs. The FT-IR characterization confirmed the successful formation of the copolymer and its nanocomposites. The influence of carbon-based nanoparticle coating is reflected in the vibrational mode with increased width and deformation compared to the bare copolymer [46–50]. Figures 3 and 4 shows the^1^H NMR and^13^C for the monomer **BPy** and polymer **PPyTri**.

### 3.4. Morphology analysis

**Figure 5.**
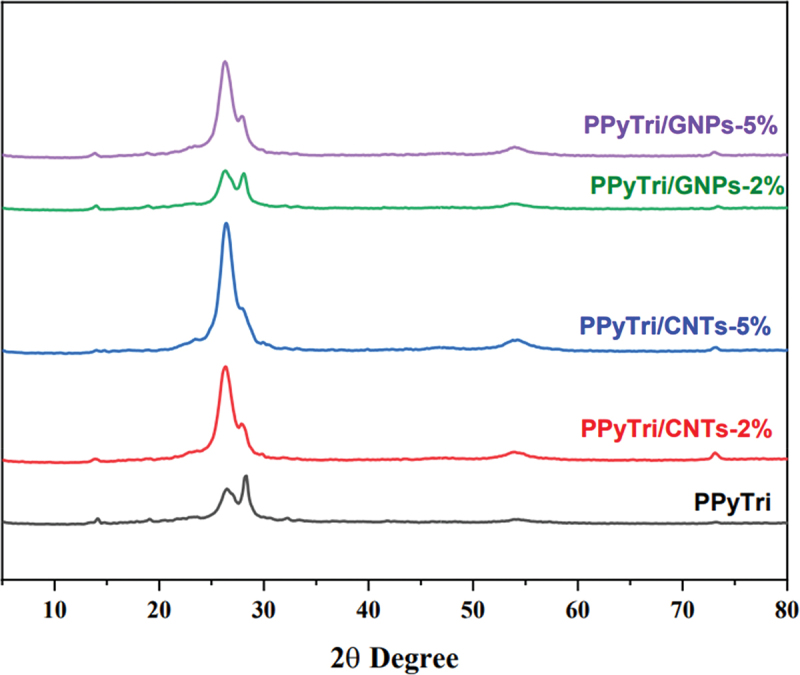
PXRD diffraction patterns for the pure copolymer PPyTri and its nanocomposites.

The surface morphologies of the synthesized copolymer and its nanocomposites were investigated by powder XRD (XRD), as shown in Figure 5. The PXRD patterns of the pure copolymer, **PPyTri**, revealed a semi-crystalline structure with a small peak at 14º and a diffraction peak (110) at 2θ = 26º. These results indicate intermolecular π–π stacking of the parallel and perpendicular periodicities for the polymer chains [51].

The spectra for the **PPyTri/CNTs-5%**, **PPyTri/CNTs-2%**, **PPyTri/GNPs-5%**, and **PPyTri/GNPs-2%** nanocomposites show bands at 26º, 28º, 54º, and 73º, attributed to the (002), (111), (220), and (400) planes of graphite. The peak intensity increased steadily as the MWCNT or GNP contents rose from 2 to 5%. Thus, the filler was effectively integrated into the copolymer molecules and dispersed throughout the polymer matrix [52–58]. The diffraction spectra showed that the additional carbon-based nanostructure impacted the nanocomposite crystallinity. With an increased MWCNT or GNP loading, the characteristic diffraction peaks became more distinct, confirming the crystalline nature of the nanocomposites.

**Figure 6.**
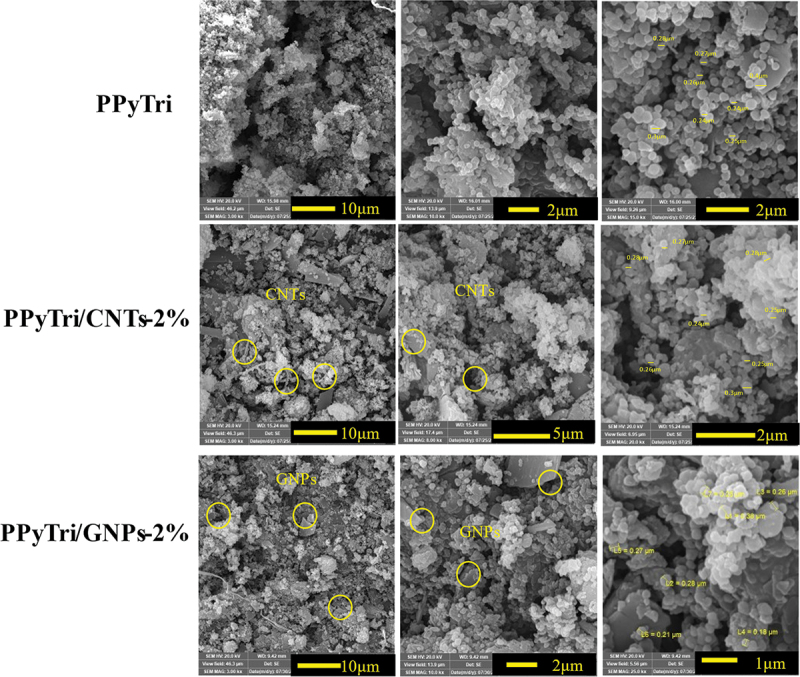
SEM images of the pure copolymer and its nanocomposites **PPyTri**, **PPyTri/CNTs-2%**, and **PPyTri/gnps-2%**.

The SEM was utilized for three designed polymers (**PPyTri, PPyTri/CNTs-2%**, and **PPyTri/GNPs-2%**) with various magnifications from 3–25 Kx to better understand the impact of MWCNTs and GNPs on the modified copolymer surface as illustrated in Figure 6. The SEM images revealed a porous network of interconnected nanofibers. The pure copolymer **PPyTri** showed a porous network of interconnected nanofibers with diameters ranging from 200–380 nm. While the surface morphology of the nanofibers was smooth, there were some visible striations along their length. The magnified nanofibers reveal their intricate and interconnected network structure.

The surface study of the nanocomposite **PPyTri/CNTs-2%** revealed a dense network of organic polymer fibers containing MWCNTs embedded throughout the micrographs. The polymer fibers exhibited a smooth surface morphology with visible striations along their length. The MWCNTs were dark, rod-like structures with diameters ranging from 20–50 nm and distributed within the polymer matrix. The **PPyTri/GNPs-2%** micrograph showed that GNPs were incorporated into the organic polymer matrix. The fact that the GNPs were partially embedded and had surface coverage indicates interactions between the two materials, potentially influencing the composite’s properties. The wrinkled and folded morphology of the GNPs increases the surface area available for interactions with the polymer or surrounding environment [46,47,59].

**Figure 7.**
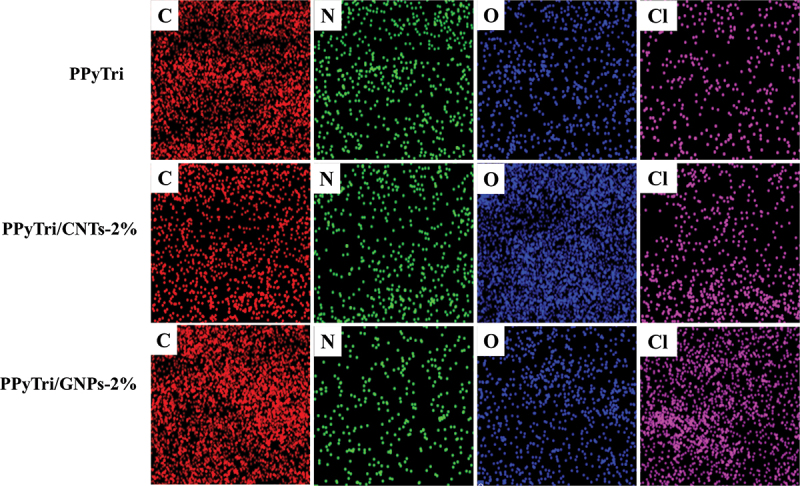
EDX elemental maps of the pure copolymer and its nanocomposites **PPyTri**, **PPyTri/CNTs-2%**, and **PPyTri/GNPs-2%**.

Further analysis of the elemental composition and homogeneity of the synthesized copolymer **PPyTri** and its nanocomposites with CNTs and GNPs was performed using energy-dispersive X-ray (EDX) mapping (Figure 7). The mapping showed the presence of C, N, O, and Cl in the unmodified **PPyTri** and its nanocomposites. The incorporated desired monomers were uniformly distributed throughout the matrix. The results of the EDX mapping, in conjunction with previous SEM observations, demonstrate the effective synthesis of the copolymer and its nanocomposites via *in situ* polymerization.

**Figure 8.**
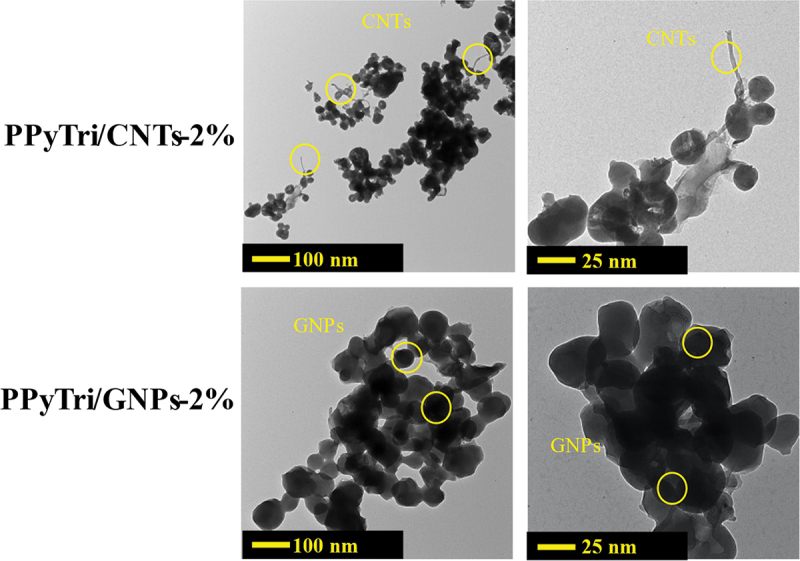
TEM images of the nanocomposites **PPyTri/CNTs-2%** and **PPyTri/GNPs-2%**.

The TEM was used to better understand how MWCNTs and GNPs disperse within the synthesized copolymer matrix. The TEM images for the **PPyTri/CNTs-2%** and **PPyTri/GNPs-2%** nanocomposites are presented in Figure 8. The images show a network of interconnected polymer fibers with a smooth surface and some discernible striations. For **PPyTri/CNTs-2%**, the MWCNTs appear as dark, tubular structures well-dispersed throughout the polymer matrix. For **PPyTri/GNPs-2%**, the GNPs appear as thin, plate-like structures with varying sizes and shapes. The TEM images agree with the SEM results of the polymer. The nanopolymer appears folded on the surface of the graphene nanosheet and forms a stacked cover. A closer look at the higher magnification inset image highlights the distributions of the polymer matrix with either MWCNTs or GNPs. The edges of some MWCNTs or GNPs appear seamlessly integrated, implying robust interfacial bonding and intermolecular interactions. This contact holds considerable promise for enhanced composite functionalities, paving the way for potential improvements in various properties. Specifically, the conductive nature of the nanomaterials coupled with the observed interfacial physical interactions suggests significant enhancements in electrical conductivity over the pure polymer[60–65].

### 3.5. Thermal study

**Figure 9.**
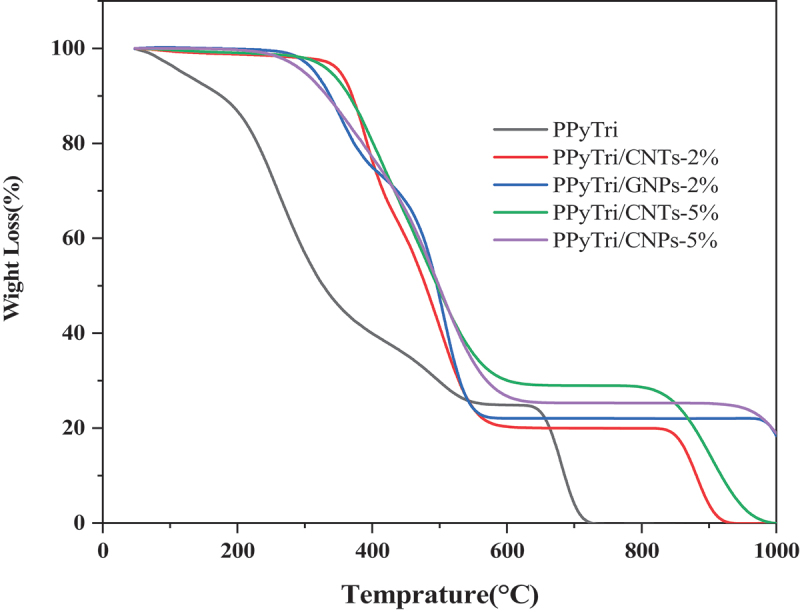
TGA for the pure copolymer PPyTri and its nanocomposites.

The thermal behaviors of the synthesized copolymers and nanocomposites were studied using thermogravimetric analysis (TGA) with temperatures from 25–1000 °C at a heating rate of 10 ºC/min (Figure 9). The TGA curve of the polymer **PPyTri** showed it underwent three stages of weight loss. The first stage was observed between 25 and 190 °C due to water and moisture loss. The second and third stages occurred between 190 and 640 °C and 640 and 720 °C, respectively, and are attributed to the degraded polymer backbone [62]. When a fixed loading of either GNPs or MWCNTs was introduced into the copolymer, the nanocomposites increased the thermal stability, which shifted both the first and second stages. The change in the first stage suggests the nanocomposites are not hygroscopic [63], while the second stage change indicates the enhanced thermal stability from ether GNPs or CNTs [66–68].

Table 1 comprehensively compares the T_10_, T_25_, and T_50_, illustrating thermal decompositions at 10%, 25%, and 50%, respectively. The values of T_10_ show the high thermal stability for 2% MWCNTs, while the values of T_25_ and T_50_ indicate a pattern where the thermal stability increases with the loaded nanofiller. Figure 7 and Table 1 demonstrate that a greater nanofiller content increases the thermal decomposition at both 25% and 50%. From a thermal perspective, the nanocomposites loaded with GNPs had a better effect on thermal stability than the corresponding CNTs. The nanocomposite **PPyTri/CNTs-2%** showed the lowest final temperature of polymer degradation (PDT_final_), while **PPyTri/GNPs-5%** exhibited the largest values at both degradation temperatures. The thermal analysis results agree with the electrochemical outcomes of the sensor performance, where the **PPyTri/GNPs-5%** registered the maximum response to the targeted analyte among all modified nanocomposites.

**Table 1.** Thermal behaviors of the polymer **PPyTri** and its nanocomposites.

Sample	Temperature (ºC) for various percentage decompositions percentages^a^			PDT_final_^a^
	T_10_	T_25_	T_50_	ºC
PPyTri	170	245	320	720
PPyTri/CNTs-2%	370	400	480	930
PPyTri/GNPs-2%	340	400	485	>980
PPyTri/CNTs-5%	365	417	490	980
PPyTri/GNPs-5%	330	420	500	1000

^***a***^*Values were determined by TGA at a heating rate of 10 °C min*^*−1*^.

### 3.6. Detection of mercury ions (Hg^+2^) employing PPyTri/CNTs/GCE and PPyTri/GNPs/GCE

The electroactivities of the modified sensors with **PPyTri/CNTs** or **PPyTri/GNPs** with both **2%** and **5%** nanostructure loadings were studied to detect Hg^+2^ ions on the GCE surface. Nafion supported the adhesion of the fabricated electrode as a conducting binding agent to create a thin, uniform layer on the GCE surface. It additionally worked as an adhesion between the nanocomposites and the electrode. Nafion has enhanced the electron transfer rate of the desired electrochemical sensors during I-V analysis [69,70]. The newly developed electrochemical sensor exhibited excellent performance in a phosphate buffer medium, displaying high sensitivity, a remarkably low DL, a wide LDR, and high stability with respectable reproducibility.

**Figure 10.**
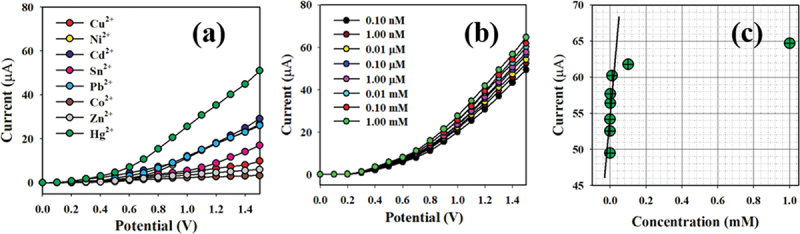
Classification of the sensor behaviors using the electrochemical (I-V) approach. (a) Selectivity estimation, (b) I-V responses based on variations in the Hg^+2^ ion concentration from low to high, and (c) calibration curve.

The initial stage of the I-V process includes investigating the selectivity of the modified senor toward Hg^+2^ ions. Various heavy metal ions at a concentration of 0.1 nM were examined under applied potentials of 0 to +1.5 V in a phosphate buffer with a pH of 7.0. As shown in Figure 10(a), Hg^+2^ ions exhibit the most significant I-V response compared to the electrochemical responses of Cu^+2^, Ni^+2^, Cd^+2^, Sn^+2^, Pb^+2^, Co^+2^, and Zn^+2^ ions. The sensor performance against Hg^+2^ ions was demonstrated with extensive concentrations ranging from 0.1 nM to 1.0 mM. The electrochemical responses presented a high distinguishability of Hg^+2^ ions throughout low and high concentrations. The calibration curve for the projected Hg^+2^ ions relative to **PPyTri/CNTs/GCE** or **PPyTri/GNPs/GCE** assesses the analytical performance, as depicted in Figure 10(c). The slope was measured to determine the sensitivity and detection limit of the Hg^+2^ sensor, recording values of 83.33 µAµM^−1^ cm^−2^ and 0.033 nM, respectively.

Figure 10(c) demonstrates the variable distribution of the current data across a linear plot spanning the concentration range of 0.1 nM to 0.01 mM at an applied potential of +1.5 V., which called as Linear Dynamic Range (LDR). There is an LDR over an extensive range of concentrations in the detection of target mercury ions using fabricated sensor probe with PPyTri/GNPs/GCE. Over the linear dynamic range, the sensor probe is started to be saturated upon addition of higher concentration until 0.1 mM. The linearity was calculated using the calibration plot drawn as current versus Hg^+2^ concentrations with a regression coefficient of r^2^ = 0.9945 under the linear dynamic range (0.10 nM to 0.01 mM).

**Figure 11.**
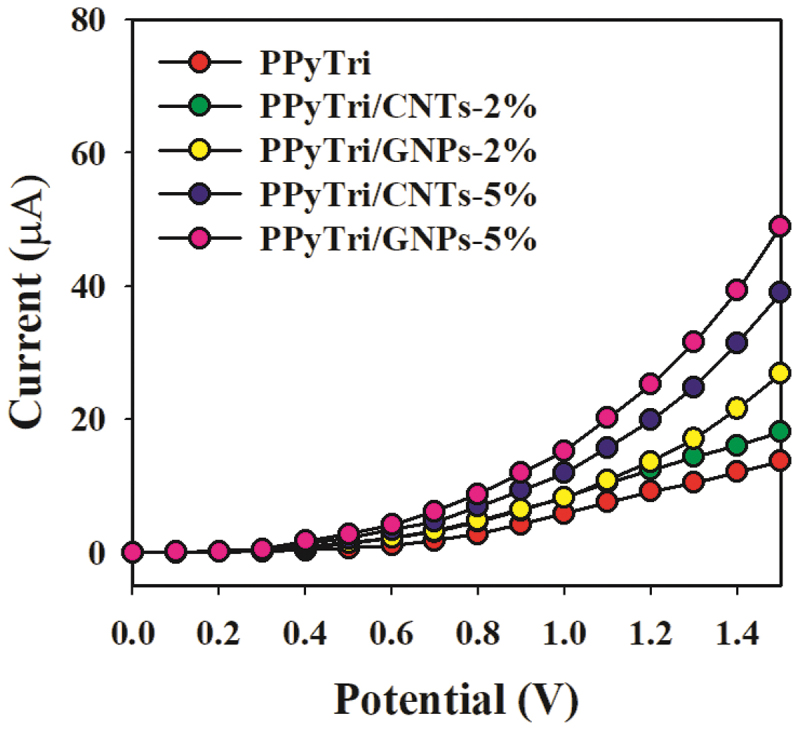
Control study executed at 0.1 µM Hg^+2^ solutions in a buffer medium with modified GCE containing **PPyTri/GNPs** or **PPyTri/CNTs (2, 5%)** nanostructure compositions.

In the control study (Figure 11), 0.1 nM Hg^2+^ solutions were utilized in a buffer medium with the adapted GCE comprising the bare copolymer **PPyTri** and the designed nanocomposites **PPyTri/CNTs-2%**, **PPyTri/GNPs-2%**, **PPyTri/CNTs-5%**, and **PPyTri/GNPs-5%**. Among the tested organic combinations, both hybrids **PPyTri/GNPs-5%** and **PPyTri/CNTs-5%**, with the highest carbon nanostructure loads, displayed the highest current response compared with the remaining investigated nanocomposites with the GCE. Between the two fabricated electrodes, **PPyTri/GNPs-5%** exhibited slightly more response to Hg^+2^ than **PPyTri/CNTs-5%** and was selected as the optimal composition for investigating Hg^2+^ ions based on the I-V approach. The expected behavior of the sensor performance is attributed to the impact of the carbon-based nanoparticles in the electron-transfer enhancement during sensing between the rich electron system and the cationic ions of Hg^+2^ [71].

**Figure 12.**
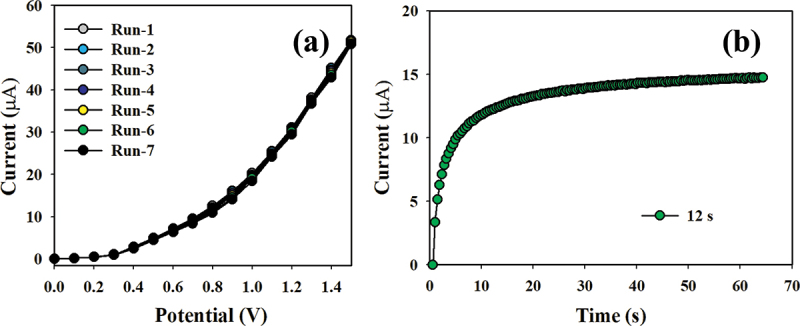
(a) Reproducibility test results and (b) response time.

Ensuring the reproducibility of electrochemical sensors is critical for credibly testing the sensor performance. The procedure used a 0.1 nM concentration of Hg^+2^ ions and was subjected to potentials ranging from 0 to +1.5 V, as illustrated in Figure 12(a). The reliability of the modified Hg^+2^ ion sensor was validated from the consistent I-V response, remaining unchanged even after washing the electrode following each of the seven replicated runs. Moreover, the response time can assess the efficiency of the formed electrochemical sensors, which is an important indicator. As shown in Figure 12(b), the projected sensor demonstrates a short response time of 12.0 s using 0.1 nM of Hg^+2^ ions.

The performance of several recent investigations for detecting Hg^+2^ ions is compared with the fabricated electrochemical sensor from this work (**PPyTri/GNPs-5%**). Table 2 shows the modified material on the electrode, the LDR, and the DL. The electrochemical sensor displays a low DL, a wide linear range, and high sensitivity. The hybrid sensor has a lower DL than our previous research using the same (I-V) method [71].

**Table 2.** Performance comparison of different electrochemical sensors for Hg^+2^ ion detection.

Modified electrode	Linear dynamic range (LDR)	Detection limit (DL)	Sensitivity [μAμM^−1^cm^−2^]	Ref.
Zn-Cd-CrO-PESNH_2_. NCs/GCE	0.1 nM to 0.1 mM	14.46 pM	0.6566	[12]
Sulfur/Co_3_O_4_/GCE	0 to 4.0 μM	0.016 μM	1027.46	[76]
NBBSH/Nafion/GCE	100.0 pM to 100.0 mM	10.0 pM	0.000949	[77]
ZIF-8 materials	0.001–50 μM	6.7 nM	-	[78]
Glycine/Ag nanoparticles	20–100 nM	17 nM	-	[79]
Zr(IV)-based MOFs	0.01 nM to 3 μM	7.3 fM	-	[80]
PMMA-SWCNT NCs/GCE	0.1 nM to 0.01 mM	55.76 pM	1.70 × 10 [2]	[71]
PPyTri/GNPs/GCE	0.1 nM to 1.0 mM	0.033 nM (33 pM)	83.33	This study

*PES: Polyethersulfone; NBBSH: (E)-N′-nitrobenzylidene-benzenesulfonohydrazide; ZIF: Zeolitic imidazolate framework; PMMA: poly(methyl methacrylate)*.

### 3.7. Potential mechanism for high-sensitivity detection of Hg(II)

The electrochemical analysis is largely contingent on the effective analyte enrichment on the electrode surface [72]. Increasing the number of enriched determinants provides a greater response signal. Hybrid materials based on carbon nanostructures (MWCNTs and GNPs) possess high conductivity with π-conjugated electron systems and charge transfer characteristics that are loaded on the fabricated electrode. The chloride counter anion and organic conjugated C3 symmetry structure enhance the system. The fabricated electrode with these properties demonstrates fast signal transduction. It further exhibits synergistic interactions between the graphene nanoparticles, **PPyTri**, and cationic Hg^+2^ ions, improving the selectivity and sensitivity of the sensing mechanism [73–75].

### 3.8. Real sample analysis

Finally, the prepared PPyTri/GNPs-5% NCs as sensor probe was implemented to measure different real samples collected from various sources, which are included in the Table 3. The validation is performed by using the recovery technique, known as standard addition approach. The acquired results are presented in Table 3, indicating the successful detection of Hg^2+^ ion in various environmental real samples by using the PPyTri/GNPs-5% NCs sensor probe with electrochemical approach. The sensor probe was found to be reliable and satisfactory, indicating its potential for various environmental monitoring applications.

**Table 3.** Validation of PPyTri/GNPs-5% NCs fabricated sensor probe using real samples by recovery method.

Real samples	Added Hg^2+^ conc. (nM)	Measured Hg^2+^ conc.^a^ by Au PPyTri/GNPs-5% NCs (nM)			Average recovery^b^ (%)	RSD^c^ (%) (*n*=3)
		R1	R2	R3		
*Industrial Effluent*	*50.00*	*50.37*	*51.37*	*49.92*	*100.11*	*0.61*
*Sea water*	*50.00*	*50.15*	*51.32*	*50.48*	*101.30*	*0.49*
*Tap water*	*50.00*	*51.33*	*49.86*	*50.08*	*100.85*	*0.65*

^*a*^*Calculations using PPyTri/GNPs-5% NCs were averaged across 3 repetitions*.

^*b*^*Measurement or calculation of Hg*^*2+*^ *concentration. (Unit: µM)*.

^c^*Accuracy in 3 replicated measurements (R1, R2, and R3) is represented by their relative standard deviation (RSD) value.*

## 4. Conclusion

The copolymer PPyTri was synthesized using a combination of triazine core hybrids and pyridinium cationic components, resulting in a C3-symmetry ionic polymer with promising structural features. The copolymer was modified with MWCNTs or GNPs with 2% and 5% loadings to enhance its electrochemical characteristics. Several characterizations were conducted to investigate the structure and morphology of the obtained nanocomposites, including IR, SEM, TEM, and PXRD. These studies provided valuable insights into the semi-crystalline nature of the polymer, the uniform distribution of nanoparticles within the polymer matrix, and the strong interactions between PPyTri and carbon nanomaterials. The results are improved mass transport and electron transfer efficiencies in electrochemical sensing applications. The electroactivity of the designed nanocomposites was studied towards different heavy metal ions with high sensitivity to Hg^+2^ ions. The GCE modified with the PPyTri/GNPs-5% NCs exhibited the greatest current response among the other combinations. The sensor performance showed excellent analytical performance in terms of sensitivity, LDR, and DL. The sensor presented consistent functioning with a short response time and ensured reproducibility.

## Data Availability

All the data has been illustrated in the manuscript text.
